# Supplementary material for: Impact of direct-acting antiviral treatment of hepatitis C on the quality of life of adults in Ukraine
Source: BMC Infect Dis. 2022 Jul 27;22:650. doi: 10.1186/s12879-022-07615-9 (PMC9330669; doi:10.1186/s12879-022-07615-9)
Supplement: Supplementary file 1 — Additional file 1: Figure S1. Mental health at study entry (baseline) and 24 weeks. Table S1. Baseline characteristics of the cohort. Table S2. Baseline characteristics, final analytic sample, and participants excluded due to missing data. Table S3. Medical Outcomes Study 20-Item Short-Form Health Survey results per domain, entry (baseline) and 24 weeks, for full cohort and stratified by HIV status. [file 12879_2022_7615_MOESM1_ESM.docx]

**Figure S1.** Mental health at study entry (baseline) and 24 weeks

**Table S1.** Baseline characteristics of the cohort

| Characteristic | HIV-  N=386 | HIV+  N=482 | Among PLHIV | | Total  N=868 |
| --- | --- | --- | --- | --- | --- |
|  |  |  | CD4<350  N=119 | CD4=>350  N=363 |  |
| Age (years) (median, interquartile range) | 40.2  (34.0-45.0) | 40.3  (36.0-43.0) | 40.0  (37.0-45.0) | 39.0  (36.0-43.0) | 39.0  (35-44.7) |
| Sex (%): Female | 138 (35.8) | 157 (32.6) | 20 (16.8) | 137 (37.7) | 295 (34.0) |
| Education beyond primary (%) | 262 (67.9) | 311 (64.5) | 77 (64.7) | 234 (64.5) | 573 (66.0) |
| Risk groups (%) |  |  |  |  |  |
| Non PWID | 40 (10.4) | 73 (15.1) | 9 (7.6) | 64 (17.6) | 113 (13) |
| PWID | 346 (89.6) | 409 (84.9) | 110 (92.4) | 299 (82.3) | 755 (87) |
| Marital status: never married (%) | 8 (2.1) | 11 (2.3) | 2 (1.7) | 9 (2.5) | 19 (2.2) |
| Median BMI (Kg/m^2^) (IQR) | 24.4  (22.1-27.2) | 23.4  (21.5-26.1) | 23.9  (21.5-25.5) | 23.3  (21.5-26.0) | 23.9  (21.6-26.5) |
| Cirrhosis % |  |  |  |  |  |
| Compensated cirrhosis | 31 (8.0) | 40 (8.30) | 16 (13.5) | 24 (6.6) | 71 (8.2) |
| No cirrhosis | 355 (92.0) | 442 (91.7) | 103 (86.6) | 339 (93.4) | 797 (91.8) |
| Infection status (%) |  |  |  |  |  |
| HCV mono-infected | 383 (99.2) | -- | -- | -- | 383 (44.1) |
| HCV/HIV | -- | 479 (99.4) | 118 (99.2) | 361 (99.5) | 482 (55.2) |
| HIV+ on ART |  |  |  |  |  |
| HCV/HBV | 3 (0.8) | 3 (0.6) | 1 (0.8) | 2 (0.6) | 3 (0.3) |
| Outcomes (N, %) |  |  |  |  |  |
| SVR12 achieved | 380 (98.5) | 451 (93.6) | 116 (97.5) | 335 (92.3) | 831 (95.7) |

**Table S2.** Baseline characteristics, final analytic sample, and participants excluded due to missing data

| **Characteristic** | **Final sample (n=857)** | **Missing (n=11)** |
| --- | --- | --- |
| Age (mean) | 40.35 (7.70) | 34.82 (6.55) |
| Age category |  |  |
| <25 yrs | 7 (0.82%) | 1 (9.09%) |
| 25-34 | 169 (19.72%) | 3 (27.27%) |
| 35-44 | 472 (55.08%) | 7 (63.64%) |
| 45-54 | 161 (18.79%) | -- |
| 55-64 | 44 (5.13%) | -- |
| >65 yrs | 4 (0.47%) | -- |
| Sex |  |  |
| Female | 294 (34.31%) | 1 (9.09%) |
| Male | 563 (65.69%) | 10 (90.91%) |
| Education level |  |  |
| Primary and below | 289 (33.72%) | 6 (54.55%) |
| Beyond primary | 568 (66.28%) | 5 (45.45%) |
| Employment status |  |  |
| Not employed | 311 (36.29%) | 5 (45.45%) |
| Employed | 546 (63.71%) | 6 (54.55%) |
| Risk group |  |  |
| PWID | 705 (86.61%) | 10 (100%) |
| Non PWID | 190 (13.39%) | -- |
| HIV Negative | 384 (44.81%) | 2 (18.18%) |
| HIV Positive | 473 (55.19%) | 9 (81.82%) |
| BMI (mean, sd) | 24.42 (4.01) | 24.82 (3.73) |
| Cirrhosis |  |  |
| Compensated cirrhosis | 71 (8.28%) | -- |
| No cirrhosis | 786 (91.72%) | 11 (100%) |
| Infection status |  |  |
| HCV mono-infected | 381 (44.46%) | 2 (18.18%) |
| HCV/HIV | 470 (54.84%) | 9 (81.82%) |
| HCV/HIV/HBV | 3 (0.35%) | -- |
| HCV/HBV | 3 (0.35%) | -- |
|  |  |  |

**Table S3.** Medical Outcomes Study 20-Item Short-Form Health Survey results per domain, entry (baseline) and 24 weeks, for full cohort and stratified by HIV status

| **Domain (mean score and 95% CI)*** | **Full cohort** | | |  | **PLHIV** | | |  | **HIV negative** | | |
| --- | --- | --- | --- | --- | --- | --- | --- | --- | --- | --- | --- |
|  | **Entry** | **24 weeks** | **% change** |  | **Entry** | **24 weeks** | **% change** |  | **Entry** | **24 weeks** | **% change** |
| Health perception | 33.5 (32.3;34.7) | 60.3 (58.8;61.7) | 86% |  | 34.1  (32.3; 36.0) | 57.7  (55.6; 59.9) | 69.9% |  | 33.2  (31.2; 35.2) | 67.9  (64.5; 70.3) | 104.4% |
| Physical functioning | 80.5 (78.9;82.1) | 89.4 (88.1;90.7) | 11% |  | 79.7  (77.5; 81.9) | 87.4  (85.5; 89.4) | 9.8% |  | 81.5  (79.1; 84.0) | 91.8  (90.0; 93.5) | 12.4% |
| Mental health | 66.9 (65.6;68.2) | 70.5 (69.4;71.6) | 5% |  | 68.4  (66.7; 70.) | 71.3  (64.8; 77.7) | 4.4% |  | 65.0  (63.1; 66.9) | 69.5  (67.8; 71.2) | 6.8% |
| Role functioning | 64.5 (62.3;66.8) | 86.5 (84.9;88.2) | 35% |  | 65.2  (62.2; 68.1) | 85.8  (83.5; 88.1) | 32.4% |  | 63.7  (60.4; 67.1) | 87.2  (84.7; 89.8) | 37.2% |
| Social functioning | 74.2 (72.1;76.2) | 84.8 (83.2;86.5) | 14% |  | 74.0  (71.3; 76.8) | 82.5  (80.1; 84.9) | 11.3% |  | 74.3  (71.4; 77.2) | 87.7  (85.5; 89.9) | 17.9% |
| Pain | 70.1 (68.2;72.0) | 89.8 (88.5;91.1) | 28% |  | 71.0  (68.4; 73.7) | 90.8  (89.2; 92.4) | 27.9% |  | 68.9  (66.1; 71.6) | 89.6  (86.5; 90.7) | 28.7% |

*Domain scores range from a low of 0 to a high of 100; higher scores indicate better health for all domains.
